# Paper-Based Biosensors for the Detection of Nucleic Acids from Pathogens

**DOI:** 10.3390/bios12121094

**Published:** 2022-11-29

**Authors:** Jiangshan Wang, Josiah Levi Davidson, Simerdeep Kaur, Andres A. Dextre, Mohsen Ranjbaran, Mohamed S. Kamel, Shreya Milind Athalye, Mohit S. Verma

**Affiliations:** 1 Department of Agricultural and Biological Engineering, Purdue University, West Lafayette, IN 47907, USA; 2 Birck Nanotechnology Center, Purdue University, West Lafayette, IN 47907, USA; 3 Department of Medicine and Infectious Diseases, Faculty of Veterinary Medicine, Cairo University, Giza 12211, Egypt; 4 Weldon School of Biomedical Engineering, Purdue University, West Lafayette, IN 47907, USA

**Keywords:** paper-based biosensors, pathogens, point-of-care testing

## Abstract

Paper-based biosensors are microfluidic analytical devices used for the detection of biochemical substances. The unique properties of paper-based biosensors, including low cost, portability, disposability, and ease of use, make them an excellent tool for point-of-care testing. Among all analyte detection methods, nucleic acid-based pathogen detection offers versatility due to the ease of nucleic acid synthesis. In a point-of-care testing context, the combination of nucleic acid detection and a paper-based platform allows for accurate detection. This review offers an overview of contemporary paper-based biosensors for detecting nucleic acids from pathogens. The methods and limitations of implementing an integrated portable paper-based platform are discussed. The review concludes with potential directions for future research in the development of paper-based biosensors.

## 1. Introduction

A pathogen is defined as an infectious organism which can be a virus, bacterium, fungus, or another microorganism that can cause disease. The detection of pathogens is key to the prevention and identification of problems related to health and safety. Traditionally, the detection of pathogens relied solely on culture-based techniques, which were considered the gold standard methods [1,2,3]. With the recent development of molecular diagnostic techniques, lab-based polymerase chain reaction (PCR) tests and next-generation sequencing are widely used due to their increased sensitivity and specificity [4,5,6]. However, these laboratory-based nucleic acid tests rely on sophisticated instruments and well-trained operators. In addition, they are prone to having longer turnaround times ranging from 1–3 days [7]. As a result, considerable effort has gone into developing rapid and cost-effective tests with equivalent or slightly lower specificity and sensitivity. These tests have the potential to be used for pathogen detection in situ by minimally trained personnel with minimal equipment.

**Figure 1:**
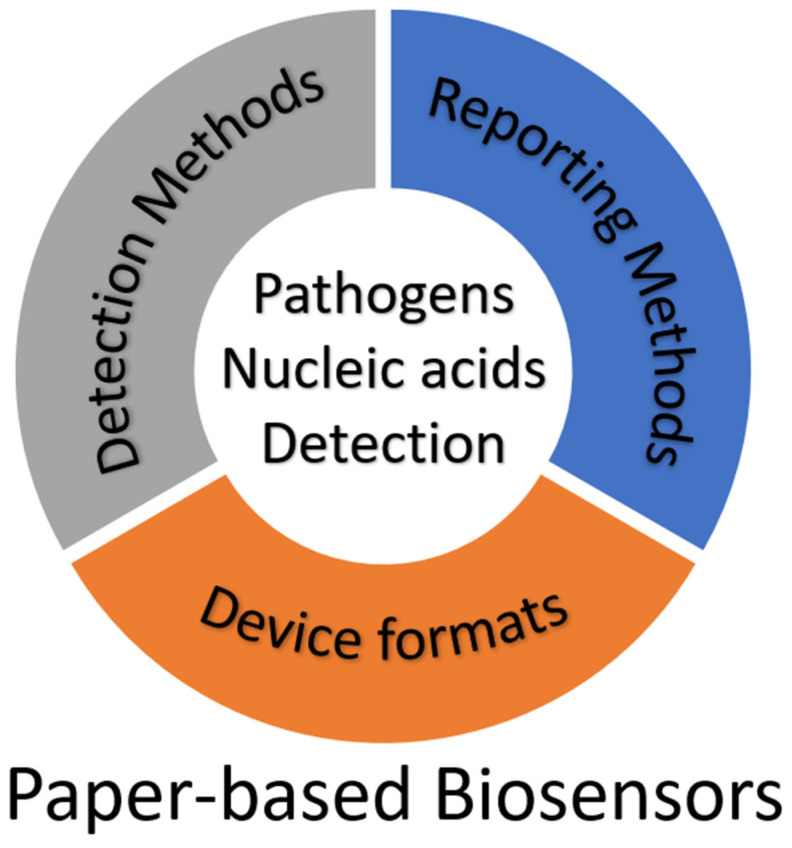
Schematic of the three essential components of paper-based biosensors for detecting nucleic acids from pathogens.

Paper-based biosensors enable the development of simple, low-cost, and flexible analytical devices [8]. They provide a number of benefits over traditional microfluidic chips, which are etched or molded with glass and polymer substrates, including a reduced cost, a simple fabrication process, strong capillary action, and good biological compatibility [9]. As a result, there is increased use of paper-based biosensors to detect analytes in clinical, food processing, and biochemical fields. Furthermore, the combination of nucleic acid testing and a paper-based platform allows for a sensitive and specific pathogen identification comparable to a lab-based test with results in less than an hour. This review will provide the reader with the literature on designs, principles, and recent progress of typical paper-based biosensors with a focus on nucleic acid detection. There are three essential components in a paper-based biosensor: detection method, reporting method, and device format (Figure 1). An integrated device comprises combinations of technologies from these three categories. As a unique characteristic of the current review, we have reviewed all the techniques used in each method. The advantages and disadvantages of each type of method/format are discussed. The summary of the currently available techniques and applications in this review could facilitate future research and development in this important but also rapidly changing field. 

## 2. Detection Method

According to its recognition mechanism, we divide the types of devices into two distinct classes: (A) structure-recognition biosensors and (B) oligonucleotide-guided biosensors. In contrast to the typical definition of pathogen structure recognition (which is targeting the outer proteins or polysaccharides of the pathogens), here, we define structure recognition as recognition of the tertiary structure of a specific nucleic acid sequence from the pathogen. The three-dimensional structures of protein-DNA and DNA/RNA-DNA complexes provide specific DNA sequence recognition. Structure-recognition biosensors include a great diversity of different assays including aptamer-based sensors, nucleic acid immunoassays, and nucleic acid hybridization sensors.

Oligonucleotide-guided biosensors require one or more guide oligonucleotides to encode target specificity. Most of the oligonucleotide-guided biosensors will amplify the target sequence by using an enzymatic process to produce multiple copies of the target from a very low copy number. Here we will introduce assays based on rolling circle amplification (RCA), recombinase polymerase amplification (RPA), loop-mediated isothermal amplification (LAMP), and clustered regularly interspaced short palindromic repeats/CRISPR-associated protein (CRISPR/Cas), highlighting their merits and demerits and reviewing their integration as paper-based biosensors for the detection of nucleic acids. 

### 2.1. Structure-Recognition Biosensors

#### 2.1.1. Aptamer-Based Sensors

Aptamers are short, single-stranded DNA or RNA (ssDNA or ssRNA) molecules that can selectively bind to a specific target. Biosensors based on aptamers are referred to as aptasensors. Given their high structural stability and capability of recovering from denaturation, aptasensors are able to detect a variety of targets such as metal ions, small molecules, proteins, pathogens, and other nucleic acids [10,11,12,13,14,15,16,17,18,19]. The main advantage of aptamers over other biorecognition molecules such as antibodies and enzymes is that they can be developed using combinatorial generation methods, such as Systematic Evolution of Ligands by Exponential Enrichment (SELEX), rather than requiring cultured organisms or animal hosts [20]. Other advantages of aptamers over other biorecognition molecules include low cost of production, custom-tailored properties, high chemical stability, high binding affinity, reproducibility, and reusability [21,22].

In the context of targeting nucleic acids, aptamers are typically used to inhibit signal transduction until a portion of the aptamer, referred to as the target recognition sequence, binds to the target. This in turn causes a structural change in the aptamer which prevents inhibition of signal transduction resulting in activation of the signal [23]. An example of this approach was first demonstrated in 2005 when Pavlov et al. utilized a nominally bound thrombin/aptamer complex to target a specific 35-base analyte DNA sequence. Upon binding, the aptamer would undergo a structural change causing it to unbind from thrombin, allowing thrombin to recover catalytic activity and produce the fluorophore, rhodamine 110, from appropriate substrates included in the reaction [24]. Using a similar enzyme activity inhibition scheme, Park et al. demonstrated that DNA polymerase activity can be blocked in the absence of a specific target DNA sequence [25]. This was achieved by attaching a target-specific sequence overhang (which nominally binds to a target-specific blocker DNA sequence) to a conserved aptamer region, which inhibited DNA polymerase. In the presence of target DNA, the blocker sequence competitively binds to the target instead of the target-specific sequence overhang. When not bound to DNA, the aptamer–overhang unit undergoes a conformational change preventing binding to polymerase, causing catalytic activity to be regained. A typical TaqMan probe can then be used to fluorometrically observe the progress of DNA polymerization. 

Certain aptamers, or regions of aptamers, can be composed of nucleic acid sequences which inherently have catalytic activity, referred to as DNAzymes or ribozymes, depending on whether the aptamer is DNA or RNA. These catalytic domains may carry out reactions that can act as a reporter. Often, DNAzymes/ribozymes contain a ligand-binding domain which may bind to other targets (in the case of this review, other nucleic acids) and subsequently regulate the activity of the catalytic domain. Taken together, the ligand-binding domain acts as a sensor for target nucleic acids, which regulates the DNAzyme/ribozyme such that a signal is produced upon binding. This subset of aptamers is referred to as an aptazyme [26]. One such example was demonstrated by Liu et al. who showed that PCR primers targeting *Helicobacter pylori* specifically designed to produce a G-quadruplex DNAzyme on the 3′ end of the PCR product could exhibit catalytic activity. The DNAzyme exhibited peroxidase-like activity to produce a colorimetric response from clear to green (using 2,2′-azino-bis(3-ethylbenzthiazoline)-6-sulfonic acid (ABTS) as the substrate) [27]. The same approach was adapted by Anantharaj et al. to target SARS-CoV-2 RNA [28]. 

Both of these aptamer-based methods, however, require intensive screening in order to identify target domains that do not interfere with the secondary structure changes required for the aptamer to inhibit and regain catalytic activity of the signal transduction element [23]. This restriction imposes major limitations on nucleic acid sequences which can serve as the target-recognition domain.

#### 2.1.2. Nucleic Acid-Modified Nanostructures

Unlike aptamers, which directly bind with a target, nanostructures such as metal–organic frameworks (MOFs), C60 or “buckyballs”, and gold nanoparticles can be modified to allow for tethering or adherence of oligonucleotides. These oligonucleotides can then act as target-recognition domains that alter the characteristics or functioning of the nanoconstructs upon binding to the target. 

**Figure 2:**
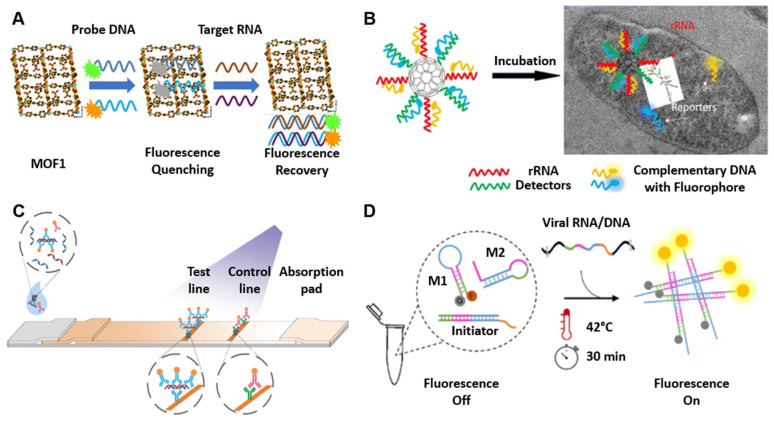
Structure-recognition biosensors. (**A**) Schematic of the 3D Cu-based metal–organic frameworks (MOF) interacting with two different fluorophore-labeled DNA probes, which can be used as effective sensing platforms for simultaneous detection of Dengue and Zika virus RNA sequences. The MOF can interact with two fluorophore-labeled DNA probes and quench the fluorescence. In the presence of Dengue virus and Zika virus RNA sequences, the DNA probes form a double-stranded DNA–RNA structure that results in fluorescence recovery. Adapted from [31] with copyright permissions from the publisher. Copyright © 2022, Elsevier. (**B**) Schematic design for the C60-rRNA detector–reporter complex, which enables bacterium detection at a specific excitation frequency. The C60-rRNA detector–reporter complex can enter the microbial cell, and when the rRNA detector hybridizes with the bacterial rRNA, the fluorophore-labeled complementary DNA is released and emits a fluorescence signal. Adapted from [32]. Copyright © The Author(s) 2017. (**C**) Schematic of hybrid capture fluorescence immunoassay (HC-FIA). The amplification-free nucleic acid immunoassay employs DNA probes that are designed to bind to the conserved regions of the SARS-CoV-2 genome and a fluorescent-nanoparticle-labeled monoclonal antibody that binds to double-stranded DNA–RNA hybrids. Adapted from [37] with copyright permissions from the publisher. Copyright © 2022, Springer Nature. (**D**) Schematic of non-enzymatic isothermal strand displacement and amplification (NISDA) assay for rapid detection of SARS-CoV-2 RNA. The viral RNA/DNA displaces the short DNA template of the initiator. The released DNA template initiates the cascade unfolding of two DNA molecular beacon structures (M1 and M2), resulting in a substantial increase in the fluorescence intensity of M1. Adapted from [46]. Copyright © The Author(s) 2021.

Metal–organic frameworks (MOFs) are a class of nanostructures that have very high porosity and a large surface area [29]. Certain MOFs can interact with DNA labeled with fluorescent probes through non-covalent and electrostatic interactions, causing the MOF to be decorated in fluorescent probes. During this interaction, the MOF quenches the fluorescence of the fluorophore via a photoinduced electron-transfer (PET) process [30]. Competition occurs when the target molecule is introduced, causing the fluorescent DNA to dissociate from the MOF and combine with the target molecule, thus regaining fluorescence. Xie et al. demonstrated that a newly synthesized and water-soluble 3D Cu-based MOF can stably interact with two different fluorescent probes, thus enabling the simultaneous detection of two different target molecules (Figure 2A) [31]. This approach was demonstrated by the simultaneous detection of Dengue and Zika virus RNA sequences, resulting in a detection time of 36 and 2 min, respectively. 

Other nanostructures such as fullerene-based carbon C60s, or “buckyballs”, can be functionalized with oligonucleotides to interact with target nucleic acids. These have the advantage of reducing the pre-treatment of the sample needed in order to be detected by enabling intracellular delivery of the C60. Cheng and Parvin demonstrate that buckyballs can be functionalized with target-specific oligonucleotides via a 3′ AminoC7 modification (Figure 2B) [32]. Fluorophores complementary to the bound oligomers can then hybridize with the functionalized C60, creating a probe complex and quenching fluorescence. The probe complex is capable of crossing the cell membrane. Similar to MOFs, competition occurs upon introduction of the target sequence, resulting in the target binding to the functionalized C60 and displacing the complementary fluorophore, thus restoring fluorescence. Cheng and Parvin demonstrate the utility of this method by developing probe complexes targeting 16S rRNA of a variety of foodborne bacteria to create a method to image pathogenic bacteria without amplification or other specialized fluorescent proteins [33].

Gold nanoparticles are another example of a nanostructure that can be functionalized or decorated with oligonucleotides to impose specificity for a specific nucleic acid target. Commonly, these biosensors utilize the surface plasmon resonance of gold nanoparticles that results in a colorimetric response upon aggregation and disaggregation. Chauhan et al. utilized two thiol-functionalized oligonucleotides complementary to conserved enteroviral nucleic acid sequences and bound to gold nanoparticles (AuNPs) via a gold–thiol surface interaction [34]. A third oligonucleotide with high affinity for enterovirus but only partial affinity to each of the two oligonucleotides bound to the AuNPs acted as a “bridging” oligonucleotide and resulted in the aggregation of AuNPs. Upon introduction of the target enterovirus sequence, the bridging oligonucleotide competitively bound with the enterovirus genome given its higher affinity, resulting in the disaggregation of AuNPs. The disaggregated AuNPs resulted in a colorimetric red shift which could be observed with the naked eye in under 60 s. Such assays are commonly paired with lateral-flow assays to allow for easy visualization of the color change. 

#### 2.1.3. Nucleic Acid Immunoassays

In many cases, lateral flow assays may be paired with antibodies to perform detection, capture, and/or reporting, and are subsequently referred to as immunoassays. Huang et al. demonstrated a AuNP-based detection within 45 min, wherein AuNPs were only functionalized with a single oligonucleotide probe, referred to as the detector probe (DP) and complementary to the target, in this case, BK polyomavirus, or BKV [35]. A second oligonucleotide probe, referred to as a capture probe (CP), was bound to biotin but was not used to functionalize a AuNP. Instead, an anti-streptavidin antibody was immobilized on the membrane, creating a test line, and streptavidin was included in the liquid phase. Anti-BSA antibodies are immobilized to capture excess AuNP–DP complexes. In the presence of BKV, the BKV genome binds to both the CP and the AuNP–DP complexes and is captured at the test line, resulting in a red line. Excess AuNP–DP complexes which do not interact with BKV are captured by the anti-BSA antibodies and form the control line. This type of assay is considered a sandwich-type immunoassay and has increased sensitivity and specificity compared to other immunoassays [36].

The antibodies used in immunoassays can vary greatly and can alter the mechanics of any given nucleic acid immunoassay. For instance, instead of using biotin-streptavidin-based reporting, Wang et al. demonstrated amplification-free detection of SARS-CoV-2 in under 60 min by capturing DNA–RNA hybrid complexes (Figure 2C) [37]. SARS-CoV-2 is a single-stranded positive-sense RNA virus ((+)-ssRNA). The inclusion of a DNA probe complementary to regions of the SARS-CoV-2 genome results in DNA–RNA hybrid complexes which can be specifically caught by S9.6 antibodies. By immobilizing S9.6 antibodies on the membrane to create a test line and also including fluorescent nanoparticle (FNP)-labeled S9.6 antibodies, when SARS-CoV-2 is present in the sample, the DNA–RNA hybrid duplexes will be caught and reported by FNP-S9.6 antibodies.

#### 2.1.4. Nucleic Acid Hybridization Sensors

In order to reduce or bypass the need for enzymes, antibodies, and nanostructures necessary for the previously discussed detection methods, substantial efforts have been invested into probing the interactions and biosensing capabilities of nucleic acids, especially the unique and sometimes complex secondary structures that have been observed [38]. 

The simplest and perhaps most broadly utilized nucleic acid secondary structure in biosensing is a simple hairpin, wherein a portion of the nucleic acids is self-complementary and folds back on itself and may or may not be accompanied by a single-stranded overhang [39]. Hairpin DNA (hpDNA) is found in many nucleic acid amplification mechanisms such as LAMP (the loop structures can be considered a hairpin), hairpin DNA-mediated isothermal amplification (HDMIA), and phosphorothioated hairpin-assisted isothermal amplification (PHAmp) [40,41,42]. In these cases, the hairpin intermediates are crucial to the robustness and sensitivity of the given assay. 

In particular, hairpins are useful when amplifying the signal, rather than the target. Two such reactions are catalyzed hairpin assembly (CHA) and hybridization chain reaction (HCR). Briefly, CHA utilizes two hairpins which are initially closed with a fluorophore and quencher on the 5′ and 3′ ends [43]. The hairpins have a partial affinity for the target molecule and partial affinity for each other. In the presence of the target (initiator strand) the first hairpin is opened via binding of the initiator. The initiator is then displaced by the binding of the second hairpin to the first hairpin, which releases the initiator to catalyze further hairpin openings. In the open state, the bound hairpins are no longer quenched and release a fluorescent signal. Similar to CHA, HCR also requires two hairpins that can coexist in a closed fashion when not in the presence of the target or initiator, but for HCR, the hairpins have internally quenched fluorophores. In the presence of the initiator, the first hairpin is opened, which results in a fluorescent signal, and an exposed region can open the second hairpin [44]. This results in a fluorescent signal and exposition of a region, which can in turn open the first hairpin. This alternating opening of hairpins continues, resulting in a nicked double helix chain with the original initiator still bound (in HCR, the initiator is not released to catalyze further hairpin openings). Both CHA and HCR are premised on toehold-mediated strand displacement, wherein an invading strand (in the case of CHA and HCR, the invading strand is the initiator) with a complementary sequence (the toehold) is able to undergo a “tug-of-war” to displace the incumbent strand. The kinetics of this displacement are controlled by the length of the toehold and can be tightly controlled.

A variety of biosensors with limits of detection as low as 17 copies/reaction have been developed utilizing both CHA and HCR with colorimetric and electrochemical reporters [39]. Typically, CHA and HCR are ideally suited for targeting very small nucleic acids, such as microRNAs (miRNA). They have, however, been adapted to detect pathogenic nucleic acids such as HIV cDNA, SARS-CoV-2, and Ebola virus with sensitivities of 18,000, 50, and 15 copies/µL (following target amplification), respectively [45,46,47]. Mohammadniaei et al. tested the non-enzymatic isothermal strand displacement and amplification (NISDA) assay with 164 clinical oropharyngeal RNA samples and successfully identified all the 65 negative samples with no false-positive rate and 60 positives out of 62 positive samples (3.2% false-negative rate) in only 30 min (Figure 2D) [46]. Both methods, however, suffer from background “leakage” stemming from impurities in the oligonucleotide hairpin synthesis, which can result in false positives [41]. This has been partially addressed by coupling signal generation and amplification strategies such as AuNP and DNA-quadruplex activity [48].

### 2.2. Oligonucleotide-Guided Biosensors 

#### 2.2.1. Rolling Circle Amplification (RCA) 

**Figure 3:**
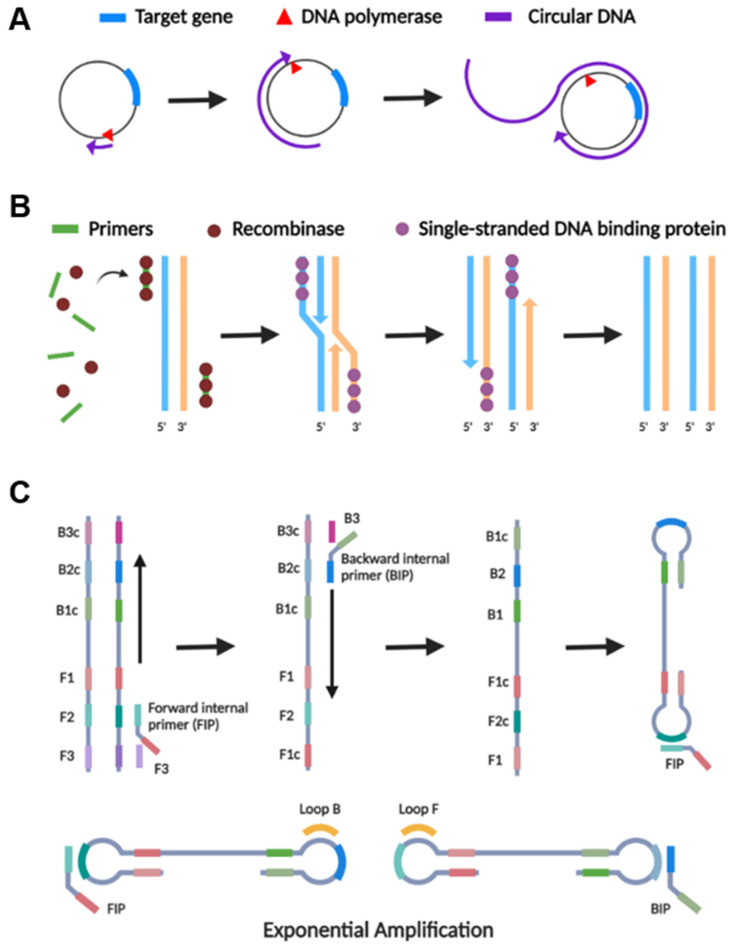
The basic schematic principles of isothermal amplification methods. (**A**) The principle of rolling circle amplification (RCA). (**B**) The principle of recombinase polymerase amplification (RPA). (**C**) The principle of loop-mediated isothermal amplification (LAMP). Created with BioRender.com.

RCA is an isothermal nucleic acid amplification process that uses a circular probe as a template to generate a lengthy concatemer ssDNA or RNA product (Figure 3A) [49]. The technology requires a DNA or RNA polymerase, homologous buffer, a relatively short DNA or RNA primer, and a circular template [50]. The reaction is usually carried out at 30–37 °C, and the amplification might take anywhere from a few hours to several days [51]. During the reaction, nucleotides are continuously added to a primer that had been annealed to a circular template by polymerase, resulting in a lengthy ssDNA containing hundreds to thousands of repeat units. Dependent on the primer and circular DNA template, RCA can detect the target with high sensitivity, ranging from aM to pM, and high specificity, distinguishing single-base pair mismatches [52]. Furthermore, RCA may be used to detect targets in clinical samples (e.g., biofluids and cells), making it an excellent method for in situ detection [53,54].

Because a circular template is required for amplification, RCA is a leading candidate for amplification of DNA molecules that exist in vivo as circular molecules, such as plasmids and certain phages [3]. However, utilizing padlock probes (PLPs), RCA can be implemented for non-circular DNA targets, yielding circular structures from linear DNA and considerably enhancing selectivity. Jain et al. describe a PLP RCA paper-based lateral flow assay for SARS-CoV-2 detection [55]. This configuration allowed for reaching a limit of detection of 1.3 × 10^6^ copies per reaction, also in complex matrices such as saliva, plant tissue extract, and bacterial culture, without any sample pre-treatment. For increased sensitivity, the reaction could be tailored to a more imaginative and powerful amplification. In conjunction with an electrochemical biosensor, RCA was reported to involve the hybridization of the amplicons with probes functionalized with redox-active labels [56]. This hybridization assay was able to detect as low as 1 copy/μL of N and S genes in less than 2 h. 

Unlike other amplification methods, RCA is a linear amplification, in which one target sequence is amplified only from its template and not from its progeny [57]. As it only occurs in a linear manner over time, it has the obvious disadvantage of a longer reaction time in comparison to other isothermal amplifications. Furthermore, whereas most studies conduct RCA at 37–40 °C for roughly 40 min, the target ligation with PLP and exonuclease treatment requires longer times and higher temperatures (e.g., 37–95 °C), increasing the overall process complexity and time to around 150 min [58]. To ensure full hybridization with the target and reduce the secondary structure of PLP, the hybridization temperature is a critical factor in the ligation reaction. Thus, an initial denaturation step of the dsDNA (usually at 95 °C) is also required for RCA [59]. Another challenge in the practical application of RCA includes nonspecific binding with a complex sample matrix due to the high molecular weight of RCA products [60]. These problems require fine-tuning the parameters, including RCA product length, order, composition, and stiffness.

The complexity and duration of the overall process, as well as the need for temperature changes, limit the application of RCA compared to other isothermal amplification methods in the development of biosensors.

#### 2.2.2. Recombinase Polymerase Amplification (RPA)

RPA is an isothermal nucleic acid amplification technology that operates at a low and consistent temperature (37–42 °C), mimicking the nucleic acid replication mechanism in cells (Figure 3B) [61]. The RPA technique utilizes a three-protein blend and two opposing primers, much like PCR, for amplification. The process initiates upon primers hybridizing to the target sequence; the recombinase initiates a strand displacement and facilitates an exponential DNA amplification reaction [62]. RPA has been a favorable choice for pathogen detection including bacterial, viral, fungal, and parasites in a point-of-care configuration to provide rapid and visual in situ pathogen detection [63,64,65,66,67,68,69]. 

A significant feature of RPA is the comparably simple design of only two primers, tolerance to inhibitors, and high specificity despite the low reaction temperature. Although PCR primers can be used directly in RPA, it is not suggested because their shorter length suggests that recombination rates are low; therefore, RPA will be slow and insensitive. Typically, 30–38 base primers are needed for efficient recombinase filament formation and RPA performance. The amplicon length of RPA is between 80 and 110 bp, thus designing RPA primers is more flexible and easier when target pathogens have high similarities within their genomes or other species [67]. 

The major advantage of RPA over most other isothermal amplification methods (e.g., LAMP, SDA, HAD, NASBA) is that it requires a much lower temperature. While other isothermal amplifications still require a constant-temperature exterior heating source, RPA can be carried out with equipment-free incubation using ambient temperature or body temperature [70,71]. However, lower operating temperatures also have drawbacks such as nonspecific amplification and primer dimerization [72]. 

RPA reagents were also shown to be stabilized in a dried form for easy transportation and storage without refrigeration on a paper chip. Ahn et al. demonstrated simultaneous detection of *E. coli*, *Staphylococcus aureus*, and *Salmonella typhimurium* using oven-dried reagent on a paper-based microfluidic device and the results are comparable to the solution-based RPA [73]. Magro et al. reported a freeze-drying method capable of stabilizing RPA reagents on a paper-based microfluidic device, demonstrating a sensitivity of 90% compared to the RT-PCR test [74]. 

The amplicon consistency is another key feature of considering RPA since it is essential to a variety of downstream applications. Many exponential isothermal amplification methods do not produce two identical copies of the target sequence, but rather an amplicon product that prompts the production of distinct amplicons in a subsequent stage [57]. For example, RPA could be coupled with next-generation sequencing (NGS), with RPA detecting a specific target and NGS for variant/mutation discrimination [75]. 

On the other hand, the complicated mix of enzymes and other additives makes it more difficult to establish RPA assays without purchasing the commercial kit from TwistDx. Although the reaction components were described in the original publication in 2006, the proprietary formulation of the TwistAmp kit is “significantly different” and is only available from the TwistDx provider, making it expensive and challenging to acquire in a global supply chain shortage setting [76,77]. On the other hand, reaction mixtures for other techniques are freely published, can be supplied from various sources, and allow researchers to create their own customized “kits”. 

Furthermore, the nonspecific amplification of RPA reported by many groups is also a concern of this technology [78,79]. The nonspecific amplification observed in RPA, however, did not occur when using the same primers in PCR reactions [78]. The addition of betaine reduced the rate of nonspecific amplification but did not eliminate it [78]. It is possible that the reaction temperature of RPA could mediate nonspecific primer binding to off-target sequences [80]. 

#### 2.2.3. Loop-Mediated Isothermal Amplification (LAMP)

The LAMP assay employs two or three pairs of specific primers recognizing 6–8 distinct regions of the target DNA sequence for a highly specific and rapid (typically within 15–30 min) amplification reaction (Figure 3C) [81]. As another isothermal amplification method, LAMP operates at a constant temperature of 60 to 65 °C and employs the *Bst* DNA Polymerase. It can also be applied for one-step RNA detection by employing a LAMP-compatible reverse transcriptase (AMV, RTx) or a DNA polymerase with strong reverse transcription activity (e.g., *Bst* 2.0, *Bst* 3.0). LAMP is an optimal candidate for point-of-care technologies because it has high specificity and it could rapidly produce amplicons (10^9^ copies of DNA segment within an hour) [82]. Since the first reported LAMP in 2000, it has been drastically refined and modified, and it is now extensively used for pathogen detection in point-of-care configurations [83,84,85]. 

In comparison with other isothermal methods of amplification, LAMP exhibits a relatively greater degree of specificity and sensitivity, quicker time of amplification, superior stability, and bypasses raw sample inhibitory challenges [86]. LAMP could operate across 2 pH units (7.3–9.3) and across 10 °C (57–67 °C) [86]. In addition, LAMP displays tolerance to untreated biological fluids such as stool, serum, or urine that commonly inhibit PCR amplification [86,87,88,89]. 

One major drawback of LAMP is the complexity of designing LAMP primers. If not correctly designed, they may deliver primer–primer or primer–nontargeted sequence interactions [90]. It is recommended to evaluate at least 2–4 complete sets of LAMP primers for optimal sensitivity and specificity before choosing a final set. Designing primer sets to differentiate short variable regions in a conserved sequence is even more difficult. Therefore, although LAMP is reported to have a great degree of specificity, it has rarely been used for genotyping or detecting point mutations. Novel methods such as allele-specific (AS)-LAMP can be used for genotyping of single nucleotide polymorphisms (SNPs) and mutations in nucleic acid sequences. Badolo et al. designed AS-LAMP primers to distinguish a single nucleotide mutation in *Anopheles gambiae s.l.*, the main malaria spreading vector [91]. The BIP primer was designed as the specific primer, with the mutation on the 3′ end of the B2 primer and an additional mismatched nucleotide to increase the specificity [91]. On the other hand, Carlos et al. had the mutation on the 3′ end of the F3 primer for single base selectivity [92]. However, both researchers mentioned the importance of an additional mismatched nucleotide at the neighboring position for single nucleotide discrimination. The reaction temperature is also critical for LAMP’s stringent single base selectivity [92]. AS-LAMP implemented on a lab-on-chip platform in the clinic is an affordable alternative to the current techniques for detecting SNPs such as NGS and ddPCR [93].

LAMP can also be customized and integrated with a variety of reporting techniques and device formats to improve its sensitivity and ease of use in applications and to fulfill a wide range of requirements. There are numerous methods for detecting the results, ranging from gel electrophoresis [94,95], observation of turbidity [95], the incorporation of colorimetric dyes [96], etc. More than a dozen strategies have been employed to ensure that LAMP can be an applicable tool in the field, including dipsticks, lateral flow assays (LFAs), lab-on-chip designs, microfluidic approaches, and integration control with smartphone technology [77,83,97]. 

#### 2.2.4. CRISPR/Cas 

Clustered regularly interspaced short palindromic repeats (CRISPR)–associated (Cas) systems, first detected in *Escherichia coli* in 1987, are a class of enzymes that degrade bacteriophage DNA after exposure to foreign genomic material and impose bacterial adaptive immunity [98]. CRISPR/Cas systems work by cleaving target DNA when a small CRISPR RNA (crRNA) anneals to the target genome [99]. To date, CRISPR systems are broadly categorized into two different classes based on their mechanism with class II CRISPR systems only requiring a single Cas protein to detect and cleave target genomes [98]. To function, CRISPR systems require a crRNA and a small trans-activating crRNA (tracrRNA). crRNAs are composed of a short complementary sequence of approximately 20 nucleotides long, referred to as the spacer, followed by a short CRISPR repeat sequence containing the protospacer adjacent motif (PAM) sequence vital to DNA targeting and cleavage. tracrRNAs are complementary to the repeat sequence at the 3′ end of the crRNA and anneal to form a dual-RNA guide, which directs the Cas protein to cleave the target DNA. By combining the crRNA and tracrRNA into a single RNA sequence, an independent single-guide RNA (sgRNA) is produced, which can be programmed to target the desired genome by altering the spacer sequence [99]. 

Certain Cas proteins (such as Cas12a and Cas14 for detection of dsDNA and Cas13 for detection of ssRNA) exhibit collateral trans-degradation of ssDNA in the vicinity after target recognition and cleavage [100]. This activity has been exploited by introducing ssDNA reporting molecules that produce a detectable signal once cleaved by Cas proteins [101]. Based on the detection methods, there are usually two types of reporting molecules: (1) ssDNA or RNA conjugated with a fluorescent reporter and quencher to the 5′ and 3′ ends [102] and (2) ssDNA or RNA conjugated with biotin and a binding partner to the 5′ and 3′ ends. Upon cleavage, the fluorescent reporter is no longer quenched and produces a fluorescent signal. For the biotin reporter molecules, upon cleavage, the binding partner is able to conjugate at the sample line of lateral flow strips, producing a positive readout.

**Figure 4:**
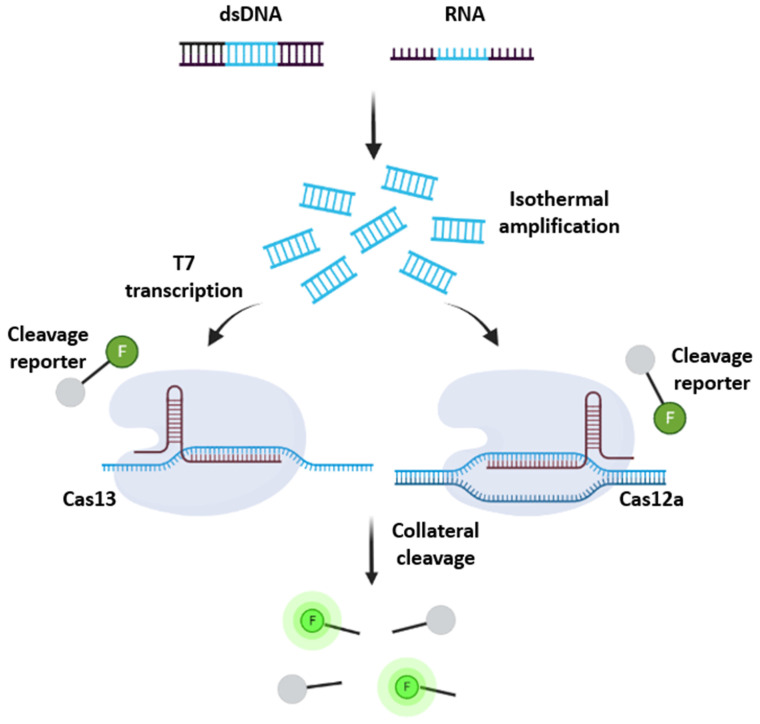
The basic schematic principle of CRISPR/Cas-based detection. DNA or RNA are amplified by isothermal amplification. Binding of the crRNA to the complementary target sequence activates the Cas enzyme and triggers collateral cleavage of quenched fluorescent reporters. Thereby, Cas13a (used in SHERLOCK) or Cas12a (used in DETECTR) indicate the presence of RNA or DNA target sequences, respectively. Created with BioRender.com.

Methods adapting various CRISPR/Cas systems such as specific high-sensitivity enzymatic reporter unlocking (SHERLOCK) and DNA endonuclease-targeted CRISPR trans reporter (DETECTR) have been developed for rapid nucleic acid detection (Figure 4) [103,104]. It was shown that these methods could detect concentrations of nucleic acid in the femtomolar range, which is 3–4 orders of magnitude worse than nucleic acid amplification techniques [105,106]. Therefore, both DETECTR and SHERLOCK typically require enrichment of the target sequence via amplification to increase the sensitivity of CRISPR/Cas-based nucleic acid detection [102]. RPA has typically been used for the amplification step due to its lower primer design complexity but also introduces significant background signals due to multiple enzymes needed, long incubation times, which can approach 120 min, and two separate reaction steps [107,108]. Consequently, loop-mediated isothermal amplification (LAMP) has been adapted to amplify and enrich the target in place of RPA. While LAMP requires the design of six primers, which increases the complexity, it can be run in a single pot, has more rapid amplification times, and utilizes only a single thermostable enzyme [108]. Regardless of which amplification strategy is chosen, the amplification step adds at least 30 min to the assay time and adds complexity to the assay. Accounting for the extraction, pre-amplification, and detection of target nucleic acids, the procedure can take between 1.5 and 6 h after sample genetic material has been extracted [103]. In the most rapid case, sample extraction accounts for 5 min, pre-amplification steps account for 30 min, and SHERLOCK detection via lateral flow detection accounts for 60 min. In the slowest case, extraction and pre-amplification can both take up to an hour each, while multiplexed fluorescent detection can take up to 4 h [103]. To decrease the maximum amount of time required, SHERLOCKv2 was developed, which utilizes Cas13 and Cas12a to produce a result in a maximum of 2 h if pre-amplification is employed [109]. Lateral flow readouts for SHERLOCK and DETECTR were accomplished by using a reporter molecule labeled with fluorescein and biotin. After incubation, a lateral flow strip is added to the reaction tube and the result is visualized after approximately 2 min [77,104]. DETECTR has demonstrated the capability of detecting SARS-CoV-2 RNA as low as 20 copies per reaction in under an hour, which accounts for a 10 min nucleic acid extraction step, 40 min pre-amplification and detection step, and a 2 min lateral flow readout step [104]. However, these established CRISPR sensing techniques include numerous manual liquid transferring steps and nucleotide extraction steps. 

Consequently, there is a push to adapt CRISPR/Cas-based biosensors towards an amplification-free assay [110]. Amplification-free CRISPR/Cas-based biosensors do not require enrichment of target nucleic acid and therefore require substantially less time to report an answer, can be run without elevated temperatures needed for isothermal amplification, and decrease the number of liquid handling steps required during the assay [110]. Liu et al. report the amplification-free detection of nucleic acids in as little as 20 min at concentrations as low as 30 molecules per µL of RNA by utilizing two nucleases in tandem, Cas13 and Csm6, along with multiple sgRNAs instead of a single sgRNA [111]. Csm6 is used to cleave the fluorescent reporter upon activation from a byproduct of the Cas13 cleavage, which allows for a fast and sensitive signal generation without amplification. Improvement of sensitivity is still needed, however, to enable the technology to be clinically relevant. Suea-Ngam et al. also devised an electrochemical CRISPR/Cas biosensor that detects cleavage of target molecules using square wave voltammetry without prior target enrichment and amplification [112]. While the device is field-deployable and cost-effective, the complexity of the specialized electrochemical device may hinder scale-up of manufacturing. Yang et al. reported a rapid and accurate CRISPR-based biosensor that utilizes the collateral effect of Cas13a and combined it with a universal autonomous enzyme-free HCR [113]. Instead of enriching the target nucleic acid, the signal amplification is realized by toehold-mediated strand displacement. The collateral effect of Cas13a releases the initiator sequence and triggers the downstream detection. With a trend towards the development of home-based devices, we expect that non-amplification methods will be adapted to function with paper-based biosensors in the future.

### 2.3. Critical Thinking in Choosing Detection Methods

The biointerface structure of the target nucleic acid and probe molecules is the key factor in determining the characteristics of molecular recognition and the performance of structure-recognition biosensors. These biosensors provide great sensitivity, rapid response, specificity, and desirable mobility, as well as simplicity and lower cost. Unlike oligonucleotide-guided biosensors, which need a sophisticated reaction matrix and the participation of several enzymes, one of the most attractive characteristics of oligonucleotide-guided biosensors is the ability to develop reagentless devices.

However, a potential drawback is the lack of universality of “structure-dependent” biosensors. As the assay is developed to recognize a specific sequence/structure of a target nucleic acid, it is most unlikely that the same recognition molecule can be employed for the detection of other pathogens. Identification and monitoring are key factors in preventing or mitigating the spread of a pathogen. Effective pathogen detection methods must be developed quickly before the consequences have a significant influence on society. Although there are technologies available for generating biorecognition molecules, such as SELEX technologies for aptamer generation, they are usually time-consuming (weeks to months) and have an unfavorable success rate (merely 30%) in discovery [114,115]. Additionally, aptamers are produced in vitro; it is uncertain whether they can function in vivo. Advances in structure-recognition biosensors require the development of a fast and effective method to produce desired biorecognition molecules or the development of a platform that could be easily modified for detecting other targets. 

Varying in primer prerequisites, time, temperature, result analysis procedures, etc., each of the oligonucleotide-guided detection techniques has its own strengths and weakness and, therefore, performs best for different purposes. The reported limit of detection of isothermal amplification methods can be found ranging from 1 to 1000 copies per reaction [116,117,118]. This might be due to the different methods used for quantifying the template concentration (e.g., UV meter, fluorometer, droplet digital PCR) or from the assay composition itself. It has been demonstrated that different reagent compositions or even primer sets will influence the assay’s sensitivity [83,119]. Dong et al. comparatively evaluated 19 sets of LAMP primers targeting different genomic regions of SARS-CoV-2 [116]. In this comparative study, since the other components of the reaction system are stable, the sensitivity and performance of the assay are mainly determined by the primers. 

Another important aspect of the assay is the reaction temperature. Detection methods such as RCA and RPA are favored because they can operate at lower temperatures (body temperature or room temperature), potentially eliminating the need for external power or heating. However, in addition to the longer reaction time and nonspecific amplification caused by the lower temperature, the reaction temperature is also insufficient for inactivation or cell lysis, necessitating a nucleic acid extraction step [117,120,121,122]. Furthermore, current point-of-care devices lack comprehensive integration. Multiple manual handling and liquid transferring processes are frequently involved. While detecting contagious pathogens, it is critical to keep workers safe and avoid the spreading of microorganisms in positive samples. The numerous manual transferring steps compromise the enclosed sample and may lead to pathogen transmission. As a result, employing a high incubation temperature, such as 60–65 °C, which is the ideal temperature for LAMP, may be beneficial in inactivating and lysing most pathogens during the reaction, including SARS-CoV-2 [123]. 

Another trend in recent advances is combining multiple techniques to achieve high specificity, efficiency, and accuracy in pathogen detection. For instance, using RPA and LAMP to increase the target sequence concentration in the original sample can lower the limit of detection of the CRISPR/Cas assay by 3–4 orders of magnitude. On the other hand, the introduction of CRISPR/Cas will eliminate the interference of nonspecific amplification products and the formation of primer-dimers during the sensing process and, therefore, does not influence the end result. To improve the sensitivity of the detection, El-Tholoth et al. developed a two-stage isothermal amplification method that utilizes both RPA and LAMP, demonstrating a 10 times better sensitivity than LAMP alone [124]. These experiments highlighted the huge potential of combining various detection methods to attain sensitivity comparable to the gold standard qPCR testing.

Reagents are usually preloaded on a paper-based biosensor. Although this approach provides convenience to the user, a reduction in assay performance is reported after lyophilization or transferring the reaction on a paper substrate [73,97,125,126]. This might be due to an overall lower catalytic activity in the filter paper assay or may be due to inactivation or loss of one or a combination of the reaction components while drying [125]. Since enzymes are used in most detection methods and are susceptible to changes over time, maintaining long-term stability of enzymes is a challenge for delivering a paper-based biosensor that maintains performance over a long time. In addition, to achieve optimal performance, uniform rehydration of the reagents is required for the paper-based biosensor. Various preservatives, paper materials, and drying techniques were tested, but no consensus was reached on which procedure or composition might be ideal [73,83,97,119,127]. By overcoming these challenges, “cold-chain storage” could be eliminated to increase the convenience and decrease the costs associated with detection devices.

## 3. Reporting Method

Signal output is another important factor that influences the overall performance of the device in terms of accuracy, sensitivity, analysis time, and assay cost. Here we review all the major reporting methods, covering electrochemical, fluorometric, and colorimetric methods that are feasible for paper-based devices. Fluorometric and electrochemical approaches typically yield better limits of detection and quantitative data. Nevertheless, interpreting the results in these formats necessitates the use of expensive or specialized equipment. Visual detection, particularly colorimetric-based detection, meets the criteria of the minimal resource requirement in point-of-care settings. Such a device is coupled with a chromogenic reporting system based on certain mechanisms such as chemical reaction, pH change, and change in the aggregation state of nanoparticles. These systems can produce a sharp color change and thus the test results can be interpreted with the naked eye. The application of each method is also constrained by other factors such as sample type, sample complexity, etc. We review recent work that has been validated using complex samples. We also summarize the characteristics of the most commonly used samples and discuss which components in those samples might interfere with the assay to provide a reference/guideline for future development based on the situation.

### 3.1. Electrochemical

The miniaturization of electrodes and their fabrication on paper paved the path for the development of cost-effective paper-based electrochemical biosensors [128]. The mechanism of electrochemical biosensing is based on using a three-electrode system, including a counter, reference, and working electrode. Each electrode serves a specific purpose to detect the analyte with a reference. To complete the circuit across the working electrode, a counter electrode is used [129]. Typically, inkjet printing techniques are used to print the electrodes on paper and make the device scalable for mass production. Paper acts as a very good base for electrochemical biosensors, as it offers high flexibility and high electrical resistance [130].

The key requirement of this detection technique is choosing the appropriate probe for hybridization with target DNA, as this can determine the test’s specificity, sensitivity, and detection limit. Other than conventional oligonucleotides, peptide nucleic acids (PNAs) have been used to achieve highly sensitive electrochemical assays [131]. PNA was first introduced by Neilson et al.; it has a peptide-like backbone of repeating N-(2-aminoethyl)-glycine units whereas nucleic acids (DNA/RNA) have a deoxyribose or ribose sugar backbone [132]. Some of the benefits of using PNA as the probe include sequence-specific binding to nucleic acids, inactivity of digestive enzymes, high chemical stability, and strong binding affinity to nucleic acids [130,133,134]. Because of its neutral nature, PNA is able to avoid nonspecific electrostatic interactions and has a high signal/noise ratio [135]. Teengam et al. used an anthraquinone-labeled pyrrolidinyl peptide nucleic acid probe developed by Vilaivan et al. for the detection of human papillomavirus (HPV) type 16 DNA on paper [134,136]. This modified probe has shown stronger binding affinity and higher specificity towards complementary DNA compared to Nielson’s PNA and DNA probes [137]. The detection limit of HPV type 16 DNA was found to be 2.3 nM (~10^9^ copies/µL) with a linear range of 10–200 nM. 

The electrochemical sensors usually use hybridization of a probe with target DNA/RNA and there is a conversion of the hybridization into an electrical signal [138]. Detection of nucleic acids using electrochemical methods on paper has been conducted in diverse ways, employing different methods for a signal readout/transduction. The voltammetric readings are the most commonly used to sense hybridization [139]. Current measurements are used as readouts in voltammetric sensors as a result of redox activities in response to hybridization. For instance, the PNA–DNA duplex in Teengam et al.’s study obstructs the accessibility of electrons from the AQ label to the electrode surface, therefore, there is a decrease in current in the presence of the target [134]. Another category of transduction is amperometric measurements; here, the redox activity is measured as a result of catalytic products in response to hybridization. Khaliliazar et al. used HRP-labeled probes to capture the DNA of toxic microalgae and took chronoamperometry measurements after adding 3,3′,5,5′-Tetramethylbenzidine (TMB) and hydrogen peroxide [140]. Another method of transduction that has been used is the electrical impedance (measurement of resistance/capacitance). Teengam et al., for tuberculosis detection on paper, measured charge transfer resistance (Rct) obtained from electrochemical impedance spectroscopy (EIS) [141].

The specificity of these assays can be controlled at even a single base-pair mismatch as this detection is hybridization-based [142]. Furthermore, this method can be designed to increase the sensitivity by immobilizing the recognition element on nanostructures with a high surface area [143]. The electronic readout allows for quantification of the nucleic acid in sample [140]. The electrochemical methods have been used to sense nucleic acid with or without amplification of the target depending on the robustness and sensitivity of the method [137,141,144]. Despite all these advantages, some challenges are associated with these devices. The probe should be able to access a single strand of the nucleic acid of the pathogen and, therefore, requires a cell lysis step and nucleic acid denaturation step (if double-stranded), requiring high-temperature treatments or chemical methods prior to running the assay [140].

### 3.2. Fluorometric

The fluorescence of molecules has been employed in different ways to report the presence and absence of nucleic acid or even in their quantification (Table 1). Some reporters exhibit increased fluorescence on interaction with nucleic acids, e.g., nucleic acid intercalating dyes. Non-intercalating dyes such as Green Calcein are dependent on the chemistry of amplification techniques to establish their function as nucleic acid reporters. Other fluorescence-exhibiting molecules, for instance, nanoparticles or organic fluorescent molecules, do not interact with DNA and need to be modified with molecules that increase their specificity towards nucleic acids.

#### 3.2.1. Fluorescent Dyes

Fluorescent dyes are the simplest fluorescent reporters for nucleic acid detection, requiring no chemical modification and, therefore, making the process of fabrication of paper-based devices easy. SYBR Green I (SG) and PicoGreen (PG) are fluorescent stains for double-stranded DNA. The fluorescence or brightness of these stains increases by >1000-fold on binding with double-stranded DNA [151]. They have been extensively used for quantification and also detection of DNA in combination with DNA amplification techniques such as PCR-based assays and LAMP. SG and PG are cyanine dyes that have similar structures, and the different structural components of these stains contribute to three kinds of interactions that stabilize the stain/DNA complex: intercalation between base pairs (narrow fit of the aromatic group between base pairs coupled with van der Waals interactions with bases significantly dampens the internal motion of SG), electrostatic interaction (positively charged thiazole group and negatively charged phosphates from the DNA backbone), and the predominant interaction with the DNA minor groove (the stain enters the DNA minor groove, intercalates, and extends its arm-like propyl groups along the groove) [152]. The intercalating properties of these dyes can inhibit the amplification reaction [145]. The alternative is to add them after the reaction. However, SYBR safe I has been reported to give a good real-time amplification signal of the target over other DNA intercalating dyes such as Evagreen, SYBR safe, and SYBR gold [146].

Non-intercalating dyes do not interfere with the amplification of the target and can be directly added to the amplification reaction on paper [153]. Commonly used fluorescent dyes that have been successfully used to develop paper-based nucleic acid detection tools are Calcein Green and Hydroxynaphthol Blue (HNB) [154]. The pyrophosphate created as a byproduct of the DNA amplification binds with the magnesium that otherwise quenches the fluorescence of these dyes. As a result, the dye is no longer bound to magnesium, and the resultant magnesium/calcein complex fluoresces upon excitation [155]. HNB is an azo dye that has been used as a colorimetric indicator in biosensing [156]. A study by Seok et al. characterized HNB for its fluorescent properties with amplified DNA products [97]. These dyes have been successfully used to quantify DNA in samples with paper assays using quantitative regression analysis, unlike colorimetric readouts, which are usually employed for yes/no readouts.

The use of fluorescent dyes as reporters comes with a few disadvantages. There can be high background fluorescence because of backscattering from paper and complex samples can also have their fluorescence giving a low signal-to-background ratio [157]. As a result, if such complex samples with strong fluorescence are being measured, it is advisable to employ longer wavelength reporters. Photobleaching is another issue, and the dyes’ instability might result in false negatives. It is critical to consider the physical environment of the site where the device is being developed to avoid such situations.

#### 3.2.2. Nanoparticles

Nanoparticles offer high amenability in optimizing the detection of nucleic acids on paper, mostly in the form of lateral flow and dip-stick tests [158]. PCR has traditionally been employed in conjunction with lateral flow assays. Recently, more isothermal amplifications have been incorporated with this method, which is a step forward toward making these devices simple to use [148]. There are some advantages of using nanoparticles, for instance, they can be modified to have a highly selective interaction with the target and, on optimization, they can give a high S/N ratio [148]. Owing to the large surface area of the nanoparticles, a greater number of target-capturing molecules can be immobilized on their surface, increasing sensitivity and improving limit of detection. Unlike fluorescent dyes, nanoparticles are stable and are less susceptible to photobleaching.

Semiconductor nanoparticles have a wide range of applications and recently they have been employed as reporters in biosensing tools [148,159]. Quantum dot is one such nanoparticle, which, when illuminated by UV light, exhibits photoluminescence. The phenomenon of photoluminescence and its ability to bind to various biomolecules makes it adaptable for a fluorescent reporter.

For nanoparticles to give consistent results, they need to have batch-to-batch consistency in the size of the particles as that would affect the number of capturing molecules immobilized on them. Chemical modification adds to the complexity of the fabrication, as compared to fluorescent dyes that are used directly.

#### 3.2.3. Fluorescent Proteins/Organic Molecules

The long wavelength emissions of fluorescent proteins can help in overcoming the limitation of fluorescent dyes of interference from complex samples [160]. R-phycoerythrin (PE) is a stable fluorescent protein that is isolated from red algae. It is a natural dye that has applications as a fluorescent marker, antioxidant, and food coloring agent, and in cosmetics such as lipsticks and eyeliners [161]. Fluorescein isothiocyanate (FITC) is another organic fluorophore that has been long used as a fluorescent probe in immunoassays [162]. FITC has excitation and emission maximums at 495 and 519 nm, respectively, while PE has an excitation peak at 496 nm and a fluorescence emission peak at 578 nm [150]. They have been used to detect other contaminants and very few studies have used them for nucleic acid detection [160]. Both the organic fluorophores were used as reporters in a lateral flow assay developed by Magiati et al. for the detection of dsDNA [150]. The fluorescent proteins are non-toxic and environmentally friendly. 

### 3.3. Colorimetric

Unlike fluorescent reporters, which require a secondary light source for excitation at a specific wavelength, colorimetric reporters can be read by the naked eye, making them an attractive alternative for POC settings and minimally instrumented devices. Colorimetric reporters can be classified by their mechanism of detection. We have identified the following mechanisms for colorimetric-based detection of nucleic acids on paper matrices: pH, aggregation, nucleic acid affinity, and Mg^2+^ depletion (Table 2).

#### 3.3.1. pH

Indicators that change color at certain pH ranges have been used in a variety of fields for a long time. These compounds are weak bases or acids which react with H^+^ or OH^−^ and display different chromophoric properties in their conjugated form. Hydrogen ions are generated as a byproduct when a DNA polymerase integrates a dNTP during amplification, which is sufficient to generate a measurable pH change when DNA amplification occurs in a weakly buffered situation [96]. While the proton release is ubiquitous to all polymerase-based amplification methods, LAMP has been the preferred method for pH-based nucleic acid detection due to its high rate of product formation (>50× PCR yield) within a short period (10–15 min) [163]. Furthermore, while PCR-based amplification undergoes a background pH shift due to temperature cycling, isothermal amplification maintains a uniform background pH.

pH-based detection of nucleic acids for diagnostic purposes offers certain advantages. Firstly, reaction output can be easily determined by a clear color change (Table 2). In addition, image analysis can be easily incorporated by extracting RGB channels of the detection area, in comparison to the complex excitation and emission required for fluorescent methods. Similarly, these indicators are stable in a dried format, which provides a distinct advantage over fluorescent reporters, which often must be stored under special conditions due to their photosensitivity.

**Table 2:** Summary of colorimetric reporters and their performance.

Colorimetric Reporters	Mechanism	Color Change (Negative-Positive)	LoD (Copies ^a^ or CFU ^b^ per Reaction)	References
Phenol red	pH	Red-Yellow	50–1000 ^a^	[83,89,164]
Phenolphthalein	pH	Pink-Colorless	1.225 ^b^	[165]
Chemosensor-L	pH	Yellow-Colorless	0.5 ^b^	[166,167]
MColorpHast	pH	Green/brown-Yellow	3.36 × 10^4 a^	[168]
Crystal violet	DNA-binding	Colorless-Purple	4413 ^a^	[169]
Methylene blue	DNA-binding	Colorless-Blue	25 ^b^	[170]
Fuchsin	DNA-binding	Colorless-Purple	1–300 ^b^	[171,172]
Gold nanoparticles	Aggregation	Colorless-Red	1.806 × 10^5^–1.806 × 10^9 a^	[173,175]
Silver nanoparticles	Aggregation	Colorless-Yellow	1.55 × 10^10^–2.30 × 10^10 a^	[176]
Polystyrene latex microspheres	Aggregation	Colorless-Red/ Colorless-Blue	1.806 × 10^11 a^	[177,178]
Hydroxynaphthol blue	Mg^2+^ depletion	Violet-Blue	2.07 × 10^4 a^	[179]

LoD was converted to copies/reaction when possible. LoD values reported in terms of mass were converted to copies using the reported length of the target DNA or when a specific accession/sequence was not specified, the median genome length of the target organism on GenBank was utilized for estimating copy number. LoD values reported as CFU were not converted due to the lack of clear correlation between CFU and gDNA copies. ^a^ copies/reaction; ^b^ colony forming unit (CFU)/reaction.

However, pH-based methods are not suitable for all conditions. Due to their mechanism, these reporters are not compatible with methods such as helicase-dependent amplification, or CRISPR/oligonucleotide-based methods which do not involve a polymerization reaction. Furthermore, only indicators that operate within a similar pH range as the LAMP polymerase (~7.0–9.0) will produce a visible color change [96]. Additionally, given it is an indirect measurement, several unrelated factors can induce a false color change such as markedly colored samples such as urine [89] or oxidation of other reagents such as ammonium sulfate [83]. As a result, some complex samples must be processed through different methods such as simple filtration, dilution [83,89], or integrated extraction [164,165,167], which increases assay complexity. Furthermore, due to uneven distribution of reagents and/or samples, color change can occur on only some regions of the device or occur only faintly [83,89], resulting in poor user interpretation compared to image analysis [83]. Increasing the indicator concentration, while helpful, will also require more protonation and become inhibitory to the reaction. Another limitation of pH-based reporters is their unquantifiable nature. None of the previously published pH-based assays reported the quantification of the initial DNA copy number. Further research is required on different paper materials to enable consistent and clear color change, as well as on combining sample processing mechanisms to increase the number of samples compatible with this technique.

#### 3.3.2. DNA Binding

Indicators that can directly bind to a target molecule are an attractive alternative due to their innate specificity. Although DNA-binding reporters are often fluorescent in nature, colorimetric alternatives typically used for cell staining protocols have recently been repurposed for nucleic acid detection in diagnostics, namely crystal violet [169], fuchsin [171,172], and methylene blue [170]. These chromophores are naturally colored in solution, yet they turn colorless when their chromophore group is attacked by a substituent such as sodium sulfite (Na_2_SO_3_). However, this binding is unstable, and these dyes form a stronger bond with DNA due to base stacking and electrostatic interaction. Therefore, in the presence of DNA, they will lose the excess sodium sulfite and bind to DNA, changing from colorless to a colored solution.

Colorimetric reporters which bind to DNA offer multiple advantages. Namely, their affinity to dsDNA over ssDNA, RNA, and dNTPs reduces the chances of false positives from other nucleic acids present in the sample, or from the reaction mixture itself [180]. Furthermore, as these reporters directly bind to DNA, they can be used for establishing quantification of DNA load in samples [169], a much-needed feature for POC diagnostics. Additionally, due to their color change behavior from colorless to colored under the presence of DNA, the image analysis can be simply conducted using a grayscale image and measuring average intensity [169,170,171,172], unlike other colorimetric reporters, which change from one color to another and require splitting the image into multiple channels for analysis.

However, this method still presents some challenges. The main one is within their mechanism for maintaining the colorless solution, requiring a substituent such as sodium sulfite for binding to the chromophore group of the molecule. The amount of excess sodium sulfite must be carefully titrated beforehand to ensure it is sufficient to minimize starting/background coloration but not large enough to prevent displacement by dsDNA [169]. Given the wide composition of real samples expected on the field, the presence of nucleophiles stronger than DNA that attack the chromophoric structure of these dyes can lead to false negatives. Even other ions such as NaCl or phosphate buffer were shown to interfere with the binding of these dyes to DNA [181]. Adding chelating agents such as EDTA and/or integrating DNA extraction [170,171] were necessary to prevent interference from other sample components. Furthermore, these methods require the addition of sodium sulfite and dye after amplification has occurred, increasing the steps for the assay and complexity of usage. Fuchsin, in particular, required LAMP amplicons to be hydrolyzed with HCl before detection to expose the aldehyde group [171,172].

#### 3.3.3. Aggregation

Advances in nanotechnology have provided a new set of tools for highly sensitive and specific detection of nucleic acids. Surface plasmon resonance present in metal nanoparticles can be exploited to use them as reporter molecules. These particles can be coupled to other structures such as oligonucleotides complementary to the target sequence, manufactured with relatively long stability, and fixed to solid supports [182].

Nanoparticle aggregation offers multiple advantages for the colorimetric detection of nucleic acids. Mainly, due to their mechanism involving target complementary oligonucleotides, they are specific, being able to differentiate double and even single nucleotide mismatches in the sequence [174,176], which is particularly useful for serotyping or mutant-specific detection. Given the high specificity of the aggregation phenomenon with the hybridized target sequence, they are resistant to protein interference [176] and the degree of aggregation can be correlated to the amount of target sequence, providing quantitative or semiquantitative capabilities to the device based on color intensity [173,174,175,176] or distance traveled by solution [177,178]. Since the aggregation is dependent on the hybridization of the target to probe sequences and not the side product of another reaction, such as in detection based on pH or Mg^2+^, an amplification step is not necessary, reducing the steps and requirements for the device. Furthermore, nanoparticles have excellent optical properties, present good stability, and can be manufactured and modified in a scalable manner. These properties, essential for POC technologies, have been validated in the multiple commercial lateral flow immunoassays in the recent COVID-19 pandemic [183].

Current shortcomings for aggregation-based colorimetric detection involve their relatively poor limits of detection (LoDs), mainly in the order of 1–10 nM. Since most devices do not include a pre-amplification step in the device, the target sequence becomes a limiting reagent. In clinical samples, the target sequence is present at too low a concentration to induce any visible aggregation. While strategies for signal amplification are possible with a combination of biotinylated streptavidin and thiolated self-recruiting AuNPs [175], these require further modification and more complementary target probes, increasing assay cost and complexity. Similarly, while the nanoparticles are easily manufactured, the custom-modified oligonucleotide probes often have complex manufacturing and purification steps. The use of PNAs with positively charged lysine reduces self-aggregation and false positives, but it increases the manufacturing requirements of the ssDNA probes for each assay [174,176]. Although PNAs increase resistance to many interferents in complex samples, under the presence of high ionic salt concentrations such as >30 mM NaCl [176] or >200 mM MgCl_2_ [174], the aggregation state of the nanoparticles did not change regardless of the addition of probes and/or target DNA. As a result, this method is not suitable for samples with naturally high salt concentrations. On the other hand, the assays reviewed in this section often required a specific sequential addition of reagents, and as such the probes and nanoparticles are kept in solution and only added after the sample, increasing the need for user intervention, and disregarding one of the main advantages of paper-based devices: drying.

#### 3.3.4. Mg^2+^ Depletion

A clever method for the detection of DNA amplicons has been measuring of magnesium pyrophosphate, another byproduct of DNA amplification. Specifically, during the amplification reaction, dNTP incorporation into the nascent strand releases pyrophosphate ions, which combine with Mg^2+^ to form insoluble magnesium pyrophosphate [184]. As an insoluble product, it can be used for monitoring reaction status through turbidity; however, this approach often has poor sensitivity even with photometric instruments [179]. To monitor the concentration of Mg^2+^ more intuitively, a colorimetric dye such as hydroxynaphtol blue (HNB), which changes color from violet to blue as Mg^2+^ concentration decreases, was incorporated [184]. While HNB and similar metal ion indicators have been used in solution-based assays, obtaining a strong colorimetric response on paper has been difficult [83,97]. Seok et al. demonstrated HNB on paper did not inhibit the LAMP amplification reaction, but the dye itself disperses between the paper pores to the point where it is no longer visible [97]. To overcome this difficulty, 2% branched polyethyleneimine (PEI) was immobilized on Whatman filter paper to prevent dispersion of HNB [179]. A quantitative range of 7880–7.88 × 10^6^ copies/µL of *E. coli* DNA was detected with the paper platform. This reporter is a cost-effective and safe way to detect LAMP amplicons [179]. Furthermore, it can be used for quantitative detection when coupled to distance measurements and does not interfere with the LAMP reaction [179].

However, HNB has several shortcomings as it can be affected by the presence of chelating agents/high salt concentrations present in the sample, and the color change from violet to blue is not as clear as other colorimetric reporters. Furthermore, while there have been multiple reports of its success in solution, efforts to translate it on paper have been limited since the dye easily disperses and fails to replicate the colorimetric response [83,97,179]. When PEI was absent, the paper device developed by Hongwarittorrn et al. did not retain any of the color change even when the LAMP reaction conducted on the solution exhibited a clear contrast [179]. This necessity of modifying paper surface further adds to device complexity.

Given the failure of HNB to replicate strong color intensity seen in solution on a paper format, it is unlikely to be suitable as a colorimetric reporter for paper-based molecular diagnostics. However, we do recognize the convenience of magnesium as an easily monitorable byproduct of the reaction progress. As such, metal ion fluorescent reporters on paper such as HNB [97,185] and calcein [186] still hold potential for magnesium-based detection.

### 3.4. Critical Thinking in Choosing Reporting Methods

With different sensitivities, response times, mechanisms, and necessary equipment for result readout, each reporting method presents unique strengths and weaknesses that must be assessed based on the specific assay’s needs.

The LoD for the various reporting methods varied in several orders of magnitude from as low as 1 copy/reaction to ~10^10^ copies/reaction [135,145,169]. Despite this broad range, all three main mechanisms discussed above were able to reach LoDs below 10 copies/µL under certain conditions [89,145,170,187]. The main challenge for reporters with regards to LoD is to generate a sufficiently high S/N ratio when only trace amounts of the target sequence are present. The two main alternatives for achieving this higher signal involve either increasing the concentration of the target sequence, mainly via isothermal amplification, or amplifying the signal of the reporter molecule itself.

Electrochemical approaches or those based on metrics such as pH or Mg^2+^ concentration are indirect measurements since they reflect a change in a secondary hybridization or amplification byproduct rather than a direct indication of nucleic acids. Although this does not exclude them as feasible reporting techniques, it does require the user to evaluate the expected inhibitors or interfering molecules within the required sample matrix that may cause signal interference.

Intercalating molecules enable sensitive and specific binding to nucleic acids. Specific molecules can be chosen to bind exclusively to target dsDNA, ssDNA, RNA, and other molecules, therefore, reducing the noise generated by other molecules. However, these intercalating dyes are often added after amplification since they can delay/inhibit the reaction [188]. The choice of the reporter molecule must consider the sample which will be assayed and its compatibility with the chosen amplification technique. For instance, phenol red was ineffective in detecting Zika virus in urine given urobilin’s strong yellow color, which interfered with the reporter’s color [89]. Similarly, phenol red with plasma samples had to be diluted to reduce the effect of sample color on the detection mechanism, whereas, with fluorescent quantum dots, a similar sample (human serum) provided significantly lower interference with the reporter without pre-treatment [89,148]. The user should decide whether steps in sample preparation or post-amplification are most suitable for the proposed assay to determine the most efficient reporter.

Another factor to consider when selecting reporter methods is whether or not quantitative results are required. Colorimetric methods have a distinct advantage in their simplicity as the color change is visible to the naked eye. However, quantifying the colorimetric readout is somewhat cumbersome. The user is required to compare the color of the reaction pad to a standard color chart previously performed by known target concentrations. Color perceptions between individuals often vary leading to misinterpretation of the result. Although colorimetric readers such as LFA readers and smartphones are available, the relationships derived are often semi-quantitative as they are not linear and/or are only linear in a limited range of concentrations [169,173]. In addition, the result interpretation step also compromises the simple purpose of colorimetric assays. 

More accurate quantitative results have been achieved through electrochemical and fluorescent reporters with dynamic ranges encompassing several orders of magnitude. However, these methods require complementary circuits and/or light sources to generate signal readout, which can become expensive and too bulky for POC settings. Electrochemical sensing does provide a significant advantage based on hybridization-based sensing given that the signal development can occur within only 5 min of sample addition, whereas, in fluorescent and colorimetric cases, between 30 min and up to 2.5 h across multiple incubation steps is necessary for signal development [148,187].

## 4. Device Formats

The most common device formats are LFAs and microfluidic paper-based analytical devices (µPADs). LFAs are widely incorporated due to their simplicity and visual readout. Without any additional equipment, lateral flow strips can provide a visible readout in as little as 2 min, and, because lateral flow strips are not assay-dependent, most developers use a commercially available strip in their device/assay. The disadvantages of the conventional LFAs are also obvious, such as only detecting one target/sample in a single test and difficulty in quantifying results. The major development of µPADs was reported by the Whitesides group in 2007, where they used patterning to create flow channels as opposed to free flow through hydrodynamic pressure [189]. Therefore, µPADs require a more sophisticated design and fabrication procedure, but they also enable the device to perform more complex reactions in one step, such as sample preparation and multiplexed detection. The current obstacles of nucleic acid detection paper-based biosensors include the short shelf-life, strict storage conditions, and poor integration of these devices. With an integrated sample-to-answer perspective, we summarize accomplishments, limitations, and future challenges for the device format of paper-based biosensors. We also suggest techniques for obtaining simple devices that could be widely used. In Table 3, we summarize recent paper-based biosensors for the detection of nucleic acids from pathogens. 

### 4.1. Lateral Flow Assays (LFAs)

The LFA is a paper-based platform for detecting and semi-quantifying analytes in complex mixtures. Due to its low cost, the versatility of formats, and user-friendliness, LFA-based tests are widely utilized in point-of-care settings for the identification of specific analytes.

LFAs normally consist of a sample pad, conjugate pad, nitrocellulose membrane, and absorbent pad, all of which are mounted to a backing sheet. The sample pad is where the sample is loaded, and, in some cases, it can also function as the first stage of filtering. After loading, the sample travels across the membrane via capillary force, activating the pre-immobilized reagents in the conjugate pad. LFA can be applied with different biorecognition molecules (antibodies, aptamers, etc.) and reporting methods (colored or fluorescent NPs, latex beads) to achieve a wide range of applications. The components of the mixture are separated as they pass through the chromatography membrane. The binding reagents will bind and generate a test line and a control line as the sample goes along the device. 

**Figure 5:**
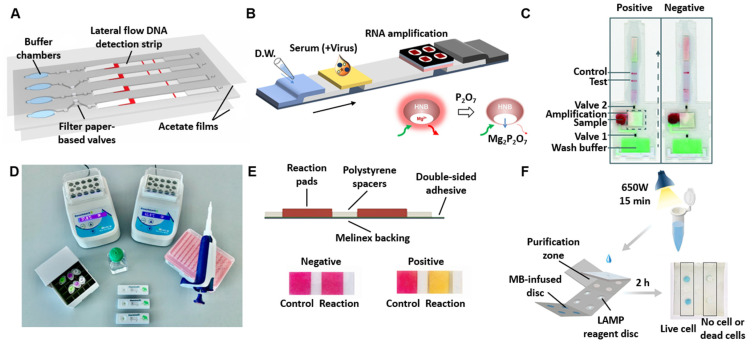
Summary of recent paper-based biosensors. (**A**) Paper-based microfluidic device that enables multiplex loop-mediated isothermal amplification (LAMP)-based detection of malaria in blood. Following the LAMP reactions in the buffer chamber, the test samples were then transferred to the lateral flow strip by pressing manually on the buffer chambers. This device can run up to three LAMP reactions at a time with a positive control to account for environmental factors and handling. Adapted from [193]. Copyright © The Author(s) 2019. (**B**) All-in-one nucleic acid testing paper chip for simultaneous detection of Zika, Dengue, and Chikungunya. The device consolidated the complete nucleic acid testing process, including sampling, extraction, amplification, and detection, onto a single paper chip. Adapted from [185] with copyright permissions from the publisher. Copyright © 2022, Elsevier. (**C**) Detection of HIV diluted in whole blood on microfluidic rapid and autonomous analytical device (microrad) with pre-dried reagents. Whole blood is deposited into the microRAAD’s sample inlet. The membrane separates HIV from blood cells, and it then migrates by capillary force to the amplification zone. Followed by amplification, the solution is released to lateral flow immunoassay (LFIA) for detection. Adapted from [197]. Copyright © The Author(s) 2019. (**D**) Equipment and consumables needed for running DNA endonuclease-targeted CRISPR trans reporter (DETECTR). The assay performs reverse-transcription LAMP (RT-LAMP) for extracted SARS-CoV-2 RNA, followed by Cas12 detection. Afterwards, a lateral flow strip is added to the reaction tube and the result is visualized after approximately 2 min. Adapted from [104]. Copyright © 2022, Springer Nature. (**E**) Schematic layout of the paper device for detecting SARS-CoV-2 in saliva. The paper-based device utilizes RT-LAMP to detect SARS-CoV-2 in whole saliva without RNA extraction. Adapted from [83]. Copyright © Elsevier 2022. (**F**) Paper-based all-in-one origami microdevice for nucleic acid amplification testing for rapid colorimetric identification of live cells for point-of-care testing. This origami paper device was successfully applied to determine the viability of foodborne pathogens by implementing a propidium monoazide treatment. Adapted from [170]. Copyright © 2022 American Chemical Society.

When it comes to qualitative assays (positive/negative outcome), LFAs are advantageous since the test result can be read in a very straightforward manner. Only a clear control line indicates that the experiment was successfully run, whereas a control line and a test line suggest a successful positive reaction. The “line” result is also colorblind-friendly. However, if the test requires quantitative analysis and high-throughput detection, it could be difficult to implement on an LFA design. Semi-quantitative analysis is possible through visual observation of the colors at the test and control lines. Quantification requires measuring the color intensity, usually via a camera along with appropriate imaging software. Strategies for multiplexing LFAs include spatially separating detection sites, using different signal reporters, and using an array of strips [196]. Reboud et al. designed a multichannel paper-based microfluidic device that enables multiplex detection of malaria in blood (Figure 5A) [193]. However, the results of a multi-zone/multi-reporter lateral flow assay are more difficult to interpret. An increase in the number of analytes of interest interferes with the identification of the closely spaced test zones [196]. Furthermore, this would result in the strip being extended (increasing the assay time) and this becoming a more sophisticated assay fabrication procedure, defeating the purpose of an LFA test. Research on simplified and low-cost methods is still needed. 

The main drawback of the conventional LFA is its poor sensitivity [158]. Several methods to improve LFA sensitivity have been used, such as sample purification, enrichment or concentration methods, enzyme-based signal enhancement, etc. Seok et al. utilized a decreasing fluidic channel (pore size of the serum loading pad > connection pad > binding pad) in the system to separate proteins or remaining cells during the lateral flow process and concentrated the target RNA on the binding pad with chitosan (Figure 5B) [185]. A doubling paper membrane served as a filter to separate human immunodeficiency virus (HIV) from whole blood via size-based separation (Figure 5C) [197]. For whole blood samples, a built-in blood separation membrane could be used prior to the LFA. Phillips et al. incorporated a PES membrane to separate the HIV virus from the whole blood sample and achieved the LoD of 2.3 × 10^7^ virus copies per mL of whole blood [197]. Methods such as SHERLOCK or DETECTR utilize an enzyme-based signal enhancement by amplifying the nucleic acid sequence via isothermal amplification techniques to enrich the target nucleic acid prior to the LFA (Figure 5D) [104]. The presence of the target sequence leads to the activation of the Cas protein, unleashing the collateral nuclease activity and degrading the dual-labeled reporter, leading to an intense test line. With the onset of nuclease activity, the intensity of the control line consequently decreases. The LFA is used for rapid visualization of the test result (approximately 2 min). Furthermore, neither the lateral flow strip nor the lateral flow cleavage reporter is specific to the target analyte. By modifying the isothermal amplification primers, this platform might readily be adapted to target additional targets of interest.

### 4.2. Microfluidic Paper-Based Analytical Devices (µPADs)

Microfluidic analytical devices, known as μPADs, use paper for liquid transport, various reactions, and reagent storage [189,198]. Capillary action inside the hydrophilic porous structure of the paper drives liquid transport in these devices, eliminating the need for an external pump to run the assay, and the liquid transport is directed by designed flow channels [199]. Various reactions, such as DNA amplification, occur within the pores, and storage of reagents is applicable when the pads containing reagents are kept in a dehydrated state. Therefore, μPADs are economically efficient devices, as compared with the chip-based microfluidic devices, and their fabrication does not usually require accessing high-tech machines and cleanrooms. On the other hand, the μPADs may have some disadvantages including a short shelf-life, strict storage conditions, and unwanted trapping of the DNA or bacterial sample within the porous structure of the paper before reaching the reaction sites, affecting the pH in the reaction mix by creating an acidic environment. Lateral flow devices are among the most basic types of μPADs that allow fluid flow in only one direction [200]. The μPADs we are referring to here differ from previous work on LFAs in the way that they use patterning to form flow channels and have the capacity to perform multiple assays simultaneously. 

The design of the μPADs can be classified into two distinct categories including 2D and 3D devices. The 2D μPADs facilitate the flow of the liquid horizontally in multiple directions. A simple form of the 2D devices consists of multiple cut PADs, separated by spacers and mounted on a backing layer [83,201]. Sometimes, a spreading mesh is also used to cover them all and facilitate the delivery of the flow among all individual paper PADs. By employing additional treatments such as folding, bending, and twisting of the paper, one can transform a 2D device into a 3D one. The 3D μPADs can provide a vertical fluid flow as well, leading to the fabrication of more complex devices suitable for multiplex and multistep assays [170,185,202]. Despite these advantages of the 3D μPADs, their fabrication and operation could be challenging. For example, if the layers of a 3D device are not accurately aligned, the liquid delivery throughout multiple layers might be adversely affected. However, this issue can be resolved by using origami techniques along with CO_2_ laser cutting instead of manually cutting and stacking multiple layers of the device [203].

In general, the fabrication methods for μPADs can be divided into two main categories: (i) patterning hydrophobic barriers within the paper and (ii) shaping methods. Several patterning techniques have been used in the literature. Among these techniques, wax printing [204,205], inkjet printing [206,207], and laser toner printing [208,209] have been high-throughput, low-cost, and simple methods. Most of the barrier-patterning methods have the drawback of the low resolution of the patterned structures. This is because the patterning agent laterally spreads within the paper [204]. A high resolution of the patterns in the paper can be achieved by using photolithography of photoresistant materials [210]. However, this method is expensive and requires sophisticated equipment and facilities. On the other hand, shaping methods such as paper cutting and laser etching provide a high resolution of the PADs, while being high-throughput and not needing any patterning reagents. These methods usually require access to a CO_2_ laser cutter or craft cutter. The cutting method may lead to the low mechanical stability of the paper and the PADs may need to be mounted on another backing material such as transparent Melinex sheets [83]. Another shaping method is embossing, which uses custom dies for cutting. A comprehensive collection of various fabrication methods and their pros and cons is available in Noviana et al. [203].

Different forms of the μPADs have been used in LAMP biosensors. For example, Davidson et al. used a simple 2D paper-based device to conduct colorimetric LAMP for the detection of SARS-CoV-2 in saliva (Figure 5E) [83]. The device consisted of two paper strips (manually cut 5 mm × 6 mm chromatography paper; Grade 222, Ahlstrom-Munksjo, Helsinki, Finland) attached to an optically clear Melinex (MELINEX^®^ 454 Polyester, Tekra, New Berlin, WI, USA) backing layer by using a double-sided adhesive tape. The paper strips were separated by 2.5 mm × 6 mm polystyrene spacers. One of the paper strips was a negative control, in which water was added instead of the LAMP primers mix, which was only included in the positive strips. A 25 μL saliva template was added to both dehydrated paper strips when running the LAMP assays. An example of the application of origami paper-based devices in performing nucleic acid testing can be seen in Trieu and Lee’s work (Figure 5F) [170]. Their device included various foldable pads, namely, splitting, purification, reaction, wicking, and dye pads. They used complementary-shaped compressing molds to emboss the channels and chambers on the cellulose paper. The devices were dipped in liquid PDMS to form the hydrophobic barriers within the paper tissue needed to guide the flow. Then, the embossed paper discs were attached to the PDMS-coated paper within the designated chambers. The splitting pad (25 × 25 mm) had a center reservoir connected to four other reservoirs, facilitating the sample delivery to the reaction sites on the purification pad. Initially, the splitting pad was folded onto the purification pad, and the wicking pad was folded underneath the purification pad. By adding a propidium monoazide-treated bacterial sample to the center hole of the splitting pad, the sample was delivered to the chitosan-coated paper discs on the purification pad where the DNA became purified. After adding the LAMP reagents onto the reaction pad, the purification pad was folded on it to perform LAMP at 65 °C. After the LAMP reaction, the origami device was unfolded and a bleaching solution was added to the chambers on the reaction pad. Finally, the dye pad was folded on the reaction pad to create a colorimetric response because of the LAMP reaction. Although the origami-fabricated μPADs could provide a good precision of the fluid delivery to the reaction sites, they do not seem to be the best option for fabricating point-of-care devices; especially when the device is going to be used by non-specialist personnel. This is because the operation of these devices includes multiple steps, folding, and liquid dispensing, which are not user-friendly for an ordinary user.

### 4.3. Integrated Design of Paper-Based Biosensors

Most of the commercialized sample-to-answer nucleic acid testing devices, such as the GeneXpert^®^ system developed by Cepheid (Cepheid, Sunnyvale, CA, USA) (https://www.cepheid.com/, accessed on 17 November 2022), Filmarray^®^ developed by Biofire (BioFire Diagnostics, Salt Lake City, UT, USA) (https://www.biofiredx.com/, accessed on 17 November 2022), and Cobis Liat^®^ developed by Roche (Roche Diagnostics, Indianapolis, IN, USA) (https://diagnostics.roche.com/, accessed on 17 November 2022), employ PCR techniques as the amplification methods with expensive thermocyclers and test analyzers can cost well above USD 20,000 [211]. A few available brands, such as the all-in-one test kit^®^ for COVID-19 diagnosis delivered by Lucira Health (https://www.lucirahealth.com/, accessed on 17 November 2022), utilize LAMP as the amplification method, providing much less expensive costs for the consumer (USD 50/test). Nearly all the commercialized test kits utilize pre-loaded reagents in liquid form, which requires considering certain instructions for their storage to keep their shelf life. Additionally, the user needs to follow several steps to complete a test and, in most cases, access to lab facilities is required. Therefore, the current state of nucleic acid testing seems to be still far from its ideal situation. 

A combination of paper-based microfluidic devices with nucleic acid detection techniques can help move toward more user-friendly, high-throughput, environment-independent, disposable, and fully integrated nucleic acid testing kits. The paper pads that hold the reagents in dry form can be placed inside a cartridge coupled with a simple fluid delivery device, such as a droplet dispenser, as a disposable unit. The user will only add the template to the disposable kit and runs the test by pressing a “start button” that triggers a self-propelled fluid delivery into the paper pads and running of the heating and detection units. The commercialization of paper-based pathogen detection biosensors for on-site nucleic acid testing by non-specialist users requires overcoming several challenges. For this purpose the future research may focus on (1) developing precise but simple fluid metering and delivery units for transferring a certain amount of DNA/RNA template to detection sites, (2) coupling the fluid delivery mechanism with the cartridges that hold paper pads to ensure a homogenous wetting of all pads located in the cartridge, (3) selecting proper materials, such as plastics and resins, to fabricate the fluid delivery mechanisms and cartridges that do not adversely affect the detection methods, (4) selecting paper types that do not interfere with the detection method or adversely affect a signal response, (5) developing imaging and signal processing units to enable a reliable readout of the results, and (6) assembling all the above components including fluid delivery, paper pads, and imaging units into a user-friendly fully integrated multiplexed testing device [212,213,214,215]. 

## 5. Conclusions and Prospects

Paper-based biosensors, an emerging bioengineering technology, bear high expectations and responsibility in the field of pathogen detection. Rapid detection of pathogens is important for preventing crises related to health, safety, and well-being. Most conventional and standard pathogen detection assays require sending samples to a centralized laboratory, slowing sample-to-answer turnaround time, and adding significant cost. This lab-based detection could be more problematic for highly contagious diseases as they spread rapidly and could lead to catastrophic events (e.g., COVID-19 pandemic). Therefore, first responders must have access to a tool that could offer rapid reliable results to enable informed decision making.

In addition to being simple, affordable, and flexible, the biosensor should also have high sensitivity and specificity that are equivalent to the gold standard test. Biosensors have developed rapidly over the past 10 years, and, despite having great potential, there have only been a few successful applications that have reached the market. In this review, we conducted a thorough evaluation of the most recent paper-based biosensor designs, principles, and recent progress with a focus on nucleic acid detection. According to the three essential components in a paper-based biosensor—detection method, reporting method, and device format—we conducted a thorough discussion to understand the advantages and limitations of each technology when designing a new biosensor. From the reviewed results, it appears that the complexity of the engineering challenges, rather than a lack of appropriate technology, is what is now preventing the development of a commercial-grade biosensor. The integration of a sample processing step, sensitive and selective detection, and sample-in answer-out into a portable device are necessary for the realization of a true in situ detection paper-based biosensor. Furthermore, as the community grows, a set of standards to evaluate the biosensor is essential. When comparing different biosensors, it can be confusing because different units are used to measure sensitivity, the performance limitations of the biosensor are not openly discussed, and the biosensors are not evaluated using a complex sample. These limitations make it challenging for other researchers to reproduce the same result and cause misleading promises and overstatement of the technology.

**Figure 6:**
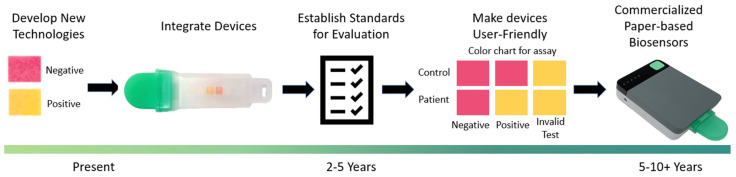
An overview of the process, challenges, and future prospects of developing commercial-grade paper-based biosensors. The review paper concluded that sufficient technologies were developed for paper-based biosensors, however, this is merely the first step in the development from the lab to the field. For the future success of paper-based biosensors and to facilitate point-of-care applications, certain aspects, such as device integration, establishing a general standard for device evaluation, and improving the user-friendliness of the device, should be addressed in the near future. To illustrate the step-by-step accomplishment, we have used data from our lab as examples of integrated devices and potential commercial prototypes. The integrated device is a cartridge that contains paper-based strips in the middle. The device under ‘commercialized paper-based biosensors’ is a heating unit that takes the cartridge and provides an output signal. These devices were built in collaboration between Purdue, Raytheon BBN, Cortex Design Inc., and Portascience Inc. (now DCNDx).

In conclusion, we identified the gaps in current paper-based biosensors and highlighted the potential for future research to facilitate point-of-care applications (Figure 6). We anticipate that the collection of all biosensor methodologies and technologies in this review could facilitate future research and development in this important but rapidly changing field.

## Figures and Tables

**Table 1:** Summary of fluorometric reporters and their performance.

Fluorometric Reporters	Type of Reporter	Target Nucleic Acid	Excitation Wavelength	LoD (Copies per Reaction)	References
SYBR Green I	Fluorescent dye	DNA	Maximum excitation 497 nm Secondary excitation peaks at 290 and 380 nm.	1–10	[145,146]
Hydroxynaphthol blue (HNB)	Fluorescent dye	DNA	530 nm	4.1 × 10^2^	[97]
Green calcein	Fluorescent dye	DNA	365 nm	25	[147]
Quantum dots	Nanoparticles	DNA	375 nm	1.12 × 10^6^	[148]
Fluorescent probes	Oligonucleotide probe	Plasmid DNA	493 nm/580 nm	10–100	[149]
R-phycoerythrin and Fluorescein isothiocyanate (FITC)	Organic fluorophore	ssDNA	496 nm and 495 nm, respectively	115	[150]

**Table 3:** Summary of paper-based biosensors for the detection of nucleic acids.

Detected Pathogen	Detection Method	Device Format	Reporting Method	Assay Time	Sample Processing	Sample Used to Determine LoD	LoD (Copies per Reaction)	Reference
SARS-CoV-2	Nucleic acid hybridization	µPADs	Electrochemical	RNA extraction time + 5 min	RNA extraction	Extracted viral RNA	2310	[187]
SARS-CoV-2	RT-LAMP; Cas12b	LFAs	Colorimetric (GNPs)	45 min	RNA extraction	In vitro-transcribed RNA in water	20	[104]
SARS-CoV-2	RT-LAMP; CRISPR–Cas12	LFAs	Colorimetric (GNPs)	90 min	RNA extraction, magnetic beads RNA concentration	Synthetic genomic standards spiked into saliva or nasopharyngeal (NP) swab	100	[77]
SARS-CoV-2	RT-LAMP	µPADs	Colorimetric (Phenol red)	60 min	Saliva dilution	Heat inactivated virus in saliva	250	[83]
SARS-CoV-2	Toehold switch	µPADs	Bioluminescent (bioluminescent reporter protein)	RNA extraction + 15 min	RNA extraction	Synthetic RNA targets in saliva	5.8 × 10^9^	[190]
Zika virus, Dengue virus	RPA; CRISPR–Cas13	LFAs	Colorimetric (GNPs)	120 min	RNA extraction	cDNA in water and urine	20	[191]
Zika virus, Dengue virus	RT-LAMP	µPADs	Fluorometric (Hydroxynaphthol blue)	60 min	RNA extraction	RNA in 1X PBS and 100% human serum	1 and 10	[192]
Zika virus, Dengue virus, Chikungunya virus	RT-LAMP	µPADs &LFAs	Fluorometric (Hydroxynaphthol blue)	60 min	RNA extraction	RNA in human serum	5	[185]
*Plasmodium* species	RT-LAMP	µPADs &LFAs	Colorimetric (Streptavidin labeled red particle)	50 min	DNA extraction, magnetic beads DNA concentration	Genomic DNA in water	32	[193]
*Plasmodium* species	RT-RPA; CRISPR–Cas12	LFAs	Colorimetric (GNPs)	70 min	DNA extraction	Genomic DNA in blood or serum	375	[194]
Hepatitis B Virus	Pyrrolidinyl peptide nucleic acid (acpcPNA)	eLFAs	Electrochemical	DNA extraction + 7 min	DNA extraction	DNA in water	4.3 × 10^6^	[137]
Hepatitis C Virus	acpcPNA	µPADs	Fluorometric (QuantiFluor^®^)	DNA extraction + 15 min	DNA extraction	Synthetic DNA oligonucleotide in water	3.0 × 10^6^	[195]

SARS-CoV-2: Severe acute respiratory syndrome coronavirus 2; RT: reverse transcription; LAMP: loop-mediated isothermal amplification; RPA: recombinase polymerase amplification; CRISPR: clustered regularly interspaced short palindromic repeats; LFA: lateral flow assays; µPADs: microfluidic paper-based analytical devices; GNPs: gold nanoparticles.

## Data Availability

No data were used for the research described in the article.
